# CPn0572, the *C*. *pneumoniae* ortholog of TarP, reorganizes the actin cytoskeleton via a newly identified F-actin binding domain and recruitment of vinculin

**DOI:** 10.1371/journal.pone.0210403

**Published:** 2019-01-10

**Authors:** Corinna Braun, Abel R. Alcázar-Román, Alexandra Laska, Katja Mölleken, Ursula Fleig, Johannes H. Hegemann

**Affiliations:** 1 Institute of Functional Microbial Genomics, Heinrich-Heine-University, Düsseldorf, Germany; 2 Eukaryotic microbiology, Institute of Functional Microbial Genomics, Heinrich-Heine-University, Düsseldorf, Germany; University of the Pacific, UNITED STATES

## Abstract

*Chlamydia pneumoniae* is one of the two major species of the *Chlamydiaceae* family that have a profound effect on human health. *C*. *pneumoniae* is linked to a number of severe acute and chronic diseases of the upper and lower respiratory tract including pneumonia, asthma, bronchitis and infection by the pathogen might play a role in lung cancer. Following adhesion, *Chlamydiae* secrete effector proteins into the host cytoplasm that modulate the actin cytoskeleton facilitating internalization and infection. Members of the conserved TarP protein family comprise such effector proteins that polymerize actin, and in the case of the *C*. *trachomatis* TarP protein, has been shown to play a critical role in pathogenesis. In a previous study, we demonstrated that, upon bacterial invasion, the *C*. *pneumoniae* TarP family member CPn0572 is secreted into the host cytoplasm and recruits and associates with actin via an actin-binding domain conserved in TarP proteins. We have now extended our analysis of CPn0572 and found that the CPn0572 actin binding and modulating capability is more complex. With the help of the fission yeast system, a second actin modulating domain was identified independent of the actin binding domain. Microscopic analysis of HEp-2 cells expressing different CPn0572 deletion variants mapped this domain to the C-terminal part of the protein as CPn0572^536-755^ binds F-actin *in vitro* and colocalizes with aberrantly thickened actin cables *in vivo*. Finally, microscopic and bioinformatic analysis revealed the existence of a vinculin binding sequence in CPn0572. Our findings contribute to the understanding of the function of the TarP family and underscore the existence of several actin binding domains and a vinculin binding site for host actin modulation.

## Introduction

*Chlamydia pneumoniae*, an obligate intracellular Gram-negative bacterium, is the most prevalent intracellular bacterial pathogen worldwide [1]. It is commonly implicated in respiratory tract infections as well as atherosclerosis, cardiovascular illnesses, and diseases of the central nervous system [2–6]. Like other *Chlamydiae*, *C*. *pneumoniae* displays a biphasic developmental cycle consisting of two metabolically and morphologically distinct developmental forms [7]. The extracellular form, referred to as an elementary body (EB), is metabolically dormant, infectious and fully capable of cellular invasion [8]. Within the confinements of a host-derived parasitophorous vacuole called an inclusion [9], EBs differentiate into reticulate bodies (RBs), which are metabolically active and non-infectious. RBs undergo several rounds of replication in a growing inclusion and eventually differentiate to infectious EBs prior to release from the host cell to initiate new rounds of infections [9]. Entry to the protective membrane-bound intracellular niche is essential to survival of *Chlamydiae* and thus represents an attractive target for disease treatment and prevention. The precise mechanism underlying chlamydial host cell invasion is still ill defined; however, it requires remodeling of the actin cytoskeleton at sites of attachment [10, 11]. The reorganization of the host actin cytoskeleton is mediated by the secretion of an actin modulating effector protein named chlamydial translocated actin recruiting phosphoprotein (TarP) through a type III secretion system [12].

Members of the TarP protein family have been identified in all sequenced *Chlamydia* species. These proteins display a number of conserved domains; however, the amino acid sequence identity is highly variable between 40 and 94% when comparing *C*. *trachomatis* L2 TarP to other TarP orthologs [11, 13, 14]. All TarP orthologs studied contain a proline-rich domain that mediates TarP oligomerization, a property required for the actin nucleation activity of *C*. *trachomatis* TarP and likely crucial for chlamydial infection [15, 16]. Furthermore all TarP family members harbor at least one and some up to three actin binding domains (ABDs) able to nucleate actin [14]. When the function of the single *C*. *trachomatis* L2 TarP ABD is obstructed during bacterial entry either through the expression of a dominant negative form of TarP that inhibits the actin nucleation ability of the wild-type TarP protein or by microinjection of anti-ABD-antibodies into the host cell, chlamydial entry is strongly inhibited [14, 17]. Thus, TarP family members play a pivotal role in actin-remodeling during chlamydial pathogenesis. Although TarP family members have a number of common characteristics required for actin modulation, an N-terminal tyrosine rich domain is only present in *C*. *trachomatis* TarP. This tyrosine rich domain becomes phosphorylated upon translocation to the host cell cytosol [12], which results in the binding of Rac GEFs, activation of Rac GTPases and ultimately Arp2/3-mediated actin remodeling [11, 18, 19]. Furthermore, focal adhesion kinase (FAK) binding motifs, vinculin binding sequences (VBSs) and ABD-independent F-actin binding domains (FABs) have to date only been identified in *C*. *trachomatis*, *C*. *muridarum* and *C*. *caviae* [13, 20–22].

The *C*. *pneumoniae* TarP ortholog CPn0572 is secreted into the host cytoplasm and associates with actin upon bacterial invasion through its single ABD region [23]. CPn0572 does not harbor the consensus TarP family FAK binding motif [20] and neither FABs nor VBSs had been identified bioinformatically [13, 21]. Thus, the possibility remained that CPn0572 actin modulating ability was solely dependent on its ABD domain. However, in this study, we show that CPn0572 harbors a second actin binding domain apart from the ABD as well as a VBS. Both domains have a profound effect on the host actin cytoskeleton. Intriguingly, the N-terminal part of CPn0572 appears to contain a domain that inhibits the ability of the ABD to colocalize with actin. Thus, the actin modulating activity of CPn0572 is driven by several domains that might affect the host actin cytoskeleton at different stages of the infection process.

## Results

### *C*. *pneumoniae* CPn0572 disrupts cytoskeleton function independently from its ABD-C domain

We have previously found that the strong actin modulatory potential of the *C*. *pneumoniae* CPn0572 protein requires the ABD-containing domain contained within amino acids 478–536 (hereinafter referred to as ABD-C for the purposes of this this manuscript) [23]. However, we now present further analysis of CPn0572 variants expressed in the model yeast *Schizosaccharomyces pombe* that pointed to the existence of additional actin modulating domains. *S*. *pombe* represents an excellent model system for the study of actin function, as cell shape, cell growth and cell division require an intact actin cytoskeleton [24–26].

**Fig 1:**
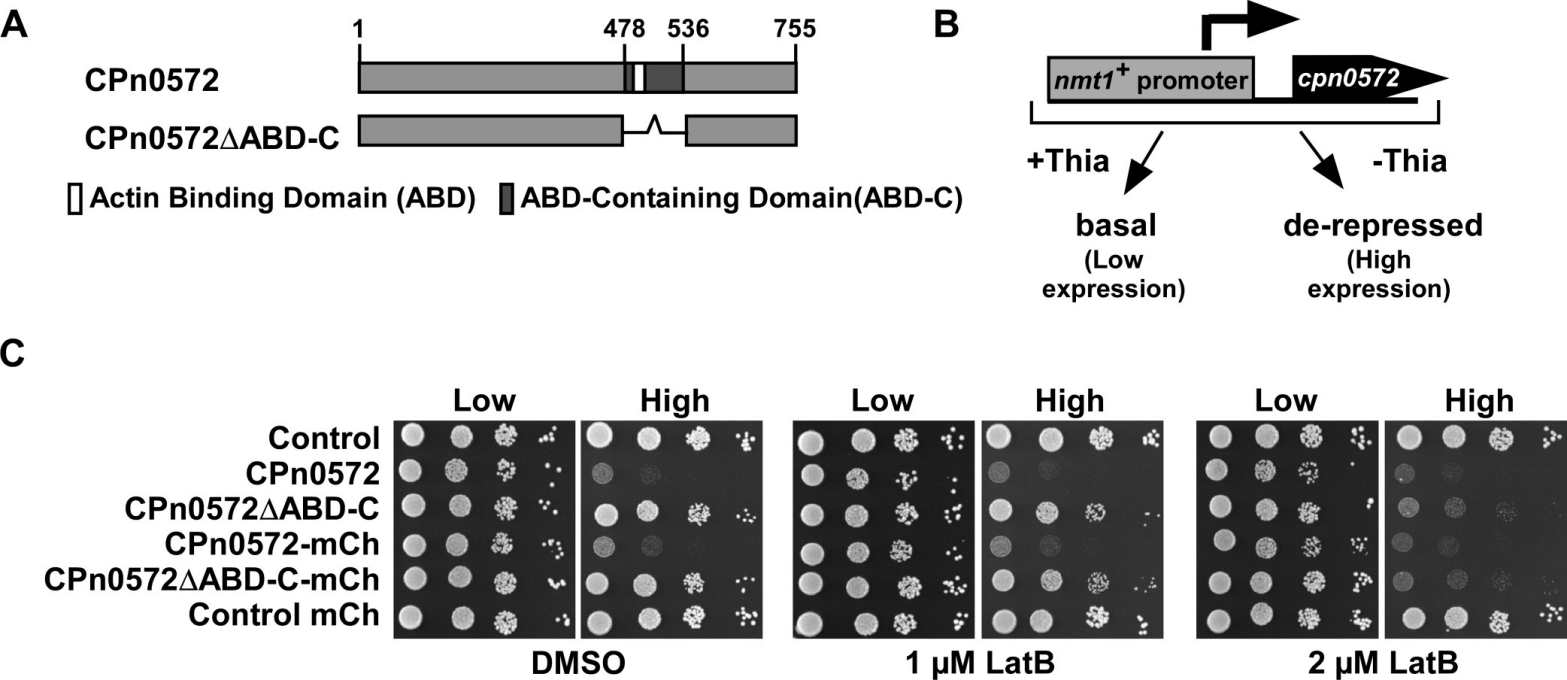
CPn0572 influences actin function in *S*. *pombe* independently of the ABD-C. **(A)** Diagrammatic representation of CPn0572 and CPn0572ΔABD-C with the location of the ABD-C (black) and ABD (white) domain. Numbers indicate amino acid positions. **(B)** Schematic representation of CPn0572 expression under control of the *nmt1*^*+*^ promoter. This promoter is repressed in the presence of thiamine (Low expression) where it shows basal expression and is de-repressed in the absence of thiamine (High expression). **(C)** Serial dilution patch test (10^4^–10^1^ cells) of a wild-type yeast strain transformed with the indicated plasmids and grown for 5 days at 30°C on plasmid selective minimal medium leading to either low expression (Low) or high expression (High) of constructs in the presence of Latrunculin B (LatB) or DMSO solvent control. Reproduced in n = 5 transformants tested per condition.

We generated plasmid-bearing *S*. *pombe* strains expressing two CPn0572 variants (Fig 1A) under the control of the *S*. *pombe nmt1*^*+*^ promoter, which is repressed in the presence of thiamine, resulting in low expression, and de-repressed in the absence of thiamine, resulting in high expression (Fig 1B and S1D Fig) [27, 28]. Throughout the text we refer to repressed as “low expression,” and de-repressed as “high expression”. Low expression of all CPn0572 variants had no effect on the growth of cells as shown via a serial dilution patch test analysis (Fig 1C). However, high expression of untagged and mCherry-tagged versions of full length CPn0572 had a drastic effect on *S*. *pombe* cell viability (Fig 1C, left panels), consistent with our previous results in the budding yeast *S*. *cerevisiae* [23]. The growth defect was dependent on the presence of the ABD-C domain as yeast cells expressing CPn0572ΔABD-C deletion constructs grew at a comparable rate to transformants harboring a control vector (Fig 1C, left panels).

Chemogenetic profiling has been used extensively in yeasts to determine genetic interactions between genes. Genes are defined as interacting genetically if the double mutant has a massively reduced fitness compared to the single mutant strains [29]. Similarly, genetic interaction is also scored if the presence of a specific chemical leads to reduced fitness of the mutant but not the wild-type strain. Thus, we next tested the viability of the transformants in the presence of the actin-depolymerizing drug Latrunculin B (LatB) at 1 μM and 2 μM, concentrations at which the actin cytoskeleton is slightly compromised but wild-type cells are able to grow normally [30, 31]. Again, high-level expression of full-length versions of CPn0572 remained a burden to cell growth in comparison to vector only controls (Fig 1C, middle panels). Low-level expression of full-length CPn0572 constructs resulted in a slight growth defect at 2 μM LatB (Fig 1C, right panels “Low”). Interestingly, high-level expression of CPn0572ΔABD-C variants resulted in a severe growth defect in the presence of LatB (Fig 1C, right panels “High”). This finding shows that high expression of CPn0572ΔABD-C has a profound negative effect on growth of the yeast colonies in the presence of LatB. We conclude that CPn0572 ABD-C compromises actin function in fission yeast independently of the ABD-C domain revealing the existence of a further actin regulatory domain or binding site.

**Fig 2:**
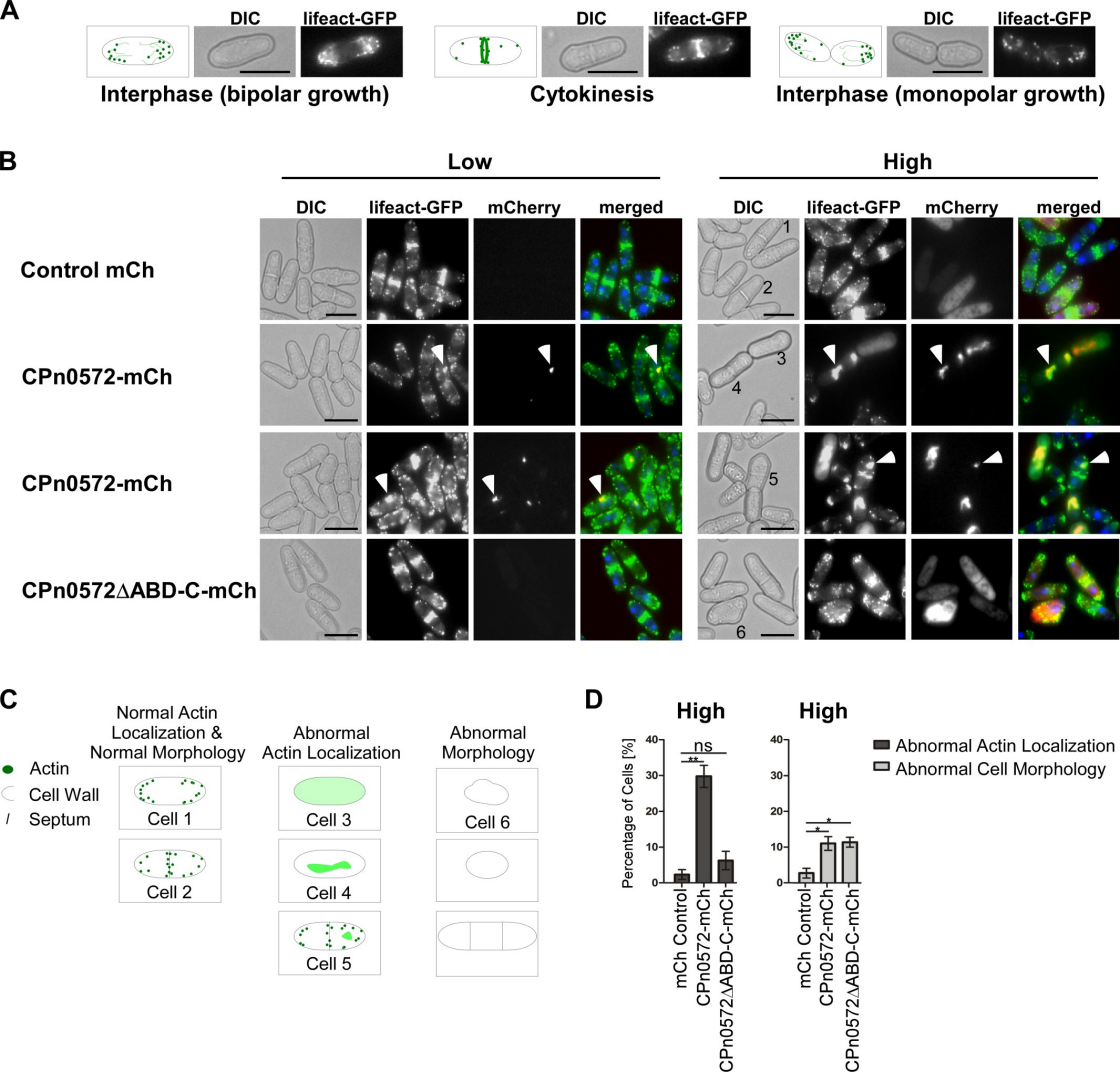
CPn0572 expression influences actin localization and morphology in *S*. *pombe*. **(A)** Actin localization during the cell cycle. Schematic representation of the Differential Interference Contrast microscopy (DIC) and lifeact-GFP images showing actin patches (green dots) and cables (green lines) localized to one or both cell ends (bipolar and monopolar growth respectively). During cytokinesis actin re-localizes to the cell middle. Bar, 5 μm **(B)** Live cell images of representative lifeact-GFP-expressing cells (F-actin is green in merged images) expressing the indicated mCherry protein variants (red in merged images). Cells were stained with Hoechst to visualize DNA (blue in merged images). Transformants were grown for 22 h under plasmid selective conditions leading to either low expression (Low) or high expression (High). White arrow heads point to selected aberrant actin structures within cells. Bars, 5 μm. **(C)** Diagrammatic representation of cells phenotypes used for quantification in D with examples labeled 1–6 shown in 2B (right panels). Normal actin localization and cell shape: Cell 1 (bipolar polarized actin localization) and Cell 2 (cytokinetic localization). Abnormal actin localization: Cell 3 (diffused actin), Cell 4 (aberrant actin structures without regular actin patches) and Cell 5 (aberrant actin structures with regular actin patches). Representative cell displaying aberrant cell morphology as depicted in schematic representation: Cell 6 (swollen cell morphology). **(D)** Quantification of cells grown under high expression of CPn0572 variants that present abnormal actin localization and abnormal cell morphology similar to the examples shown in B and S1 Fig A. n = 3 samples each representing 40–80 cells. Error bars denote standard error of the mean. Student’s t-test was used to determine statistical significance. p < 0.005 (**), p < 0.05 (*), and not significant (ns).

We next analyzed the fission yeast actin cytoskeleton in the presence of CPn0572 variants. CPn0572-mCherry or CPn0572ΔABD-C-mCherry expressing plasmids were transformed into a yeast strain containing a chromosomal copy of a gene encoding the actin probe lifeact fused to GFP, which decorates F-actin in living cells [32, 33]. This probe allowed us to visualize the three yeast actin structures: actin patches, actin cables, and the contractile ring. Cellular actin localization is cell cycle dependent (Fig 2A): in interphase, actin patches/cables are associated with one or both cell ends of the cylindrical fission yeast cell, representing monopolar or bipolar growth, respectively. After mitosis, cytokinesis requires the actin contractile ring in the cell middle for septum formation. It is also at these sites of actin-dependent cell growth that cell wall deposition takes place [34]. At low expression levels of CPn0572-mCherry 4.6% of cells showed abnormal actin localization phenotypes. The majority of these cells presented one or two slightly enlarged abnormal actin structures that differed in size to the normal actin patches that coexisted within these cells (Fig 2B, left panels 2 and 3). Interestingly, these aberrant actin structures colocalized with CPn0572-mCherry suggesting that CPn0572 presence was the cause of the abnormal actin cytoskeleton. Among cells expressing low levels of mCherry or CPn0572ΔABD-C-mCherry, less than 0.5% showed scorable defects in the yeast actin cytoskeleton (Fig 2B, left top and bottom panels, respectively).

High-level expression of CPn0572-mCherry resulted in an increased number of aberrant actin structures. Most prominently were very large actin structures that colocalized with the mCherry fluorescence signal (Fig 2B, right panels 2 and 3). 30% of cells showed an array of abnormal actin localization phenotypes including abnormal size of actin patches, diffused cytoplasmic actin or increased accumulation of actin at the contractile ring (Fig 2B, right panels 2 and 3; quantified in Fig 2D, sketched in Fig 2C). Interestingly, CPn0572-mCherry was only able to completely colocalize with actin when cells displayed very large aberrant structures (Fig 2B, right panel 3), and did not colocalize to all actin patches or septa when the cell cytoskeleton retained its classical structures (Fig 2B, right panel 3, Cell 5). Additionally, 11% of cells visualized exhibited abnormal cell morphology such as spherical, multiseptated, or deformed cells (Fig 2D, S1A Fig; sketched in Fig 2C), which are cell phenotypes associated with defects in actin cytoskeleton organization [26].

High-level expression of CPn0572ΔABD-C-mCherry resulted in a diffuse cytoplasmic distribution (Fig 2B, right bottom panels). Interestingly, even though these cells presented apparently normal actin localization without the large abnormal actin structures observed in CPn0572-mCherry expressing cells, 11.4% displayed abnormal cell morphology, including loss of polarized growth and irregular septa, consistent with the presence of a defective actin cytoskeleton (Fig 2B, right bottom panels; quantified in Fig 2D, sketched in Fig 2C). Importantly, the stronger phenotypes observed in cells expressing full length CPn0572 compared to cells expressing CPn0572ΔABD-C are not due to higher expression levels of the full length construct, as protein levels of CPn0572ΔABD-C were found to be higher (S1D Fig).

The actin cytoskeleton plays an essential part in the deposition of cell wall material [34]. To examine if CPn0572 expression has an impact in cell wall integrity, we examined the areas in which new cell wall deposition occurs by staining cells with the fluorescent dye Calcofluor white. Control cells exhibited the typical staining at the cell periphery, with slight increase at the tip of cells undergoing bipolar growth and strong staining at the septa, where new cell wall is deposited during cytokinesis (S1B Fig, top panels). Interestingly, in cells expressing CPn0572-mCherry, a dramatic buildup of cell wall material was detected at the cell middle in 24.6% of cells (S1B Fig, bottom panels; quantified in S1C Fig), consistent with an altered actin cytoskeleton.

Together our analysis of CPn0572 function in *S*. *pombe* demonstrated that the yeast actin cytoskeleton was modulated by the chlamydial protein leading to increased sensitivity to LatB and massive defects in cell morphogenesis and septum formation. Similar results, albeit with a less severe phenotype in actin morphology, resulted from CPn0572ΔABD-C expression in yeast. Thus, actin modulation is not solely dependent on the ABD-C domain of CPn0572. We therefore generated a large number of different CPn0572 variants to identify new actin binding domains.

### The C-terminus of CPn0572 mediates localization to the human actin cytoskeleton independently from the ABD-C

**Fig 3:**
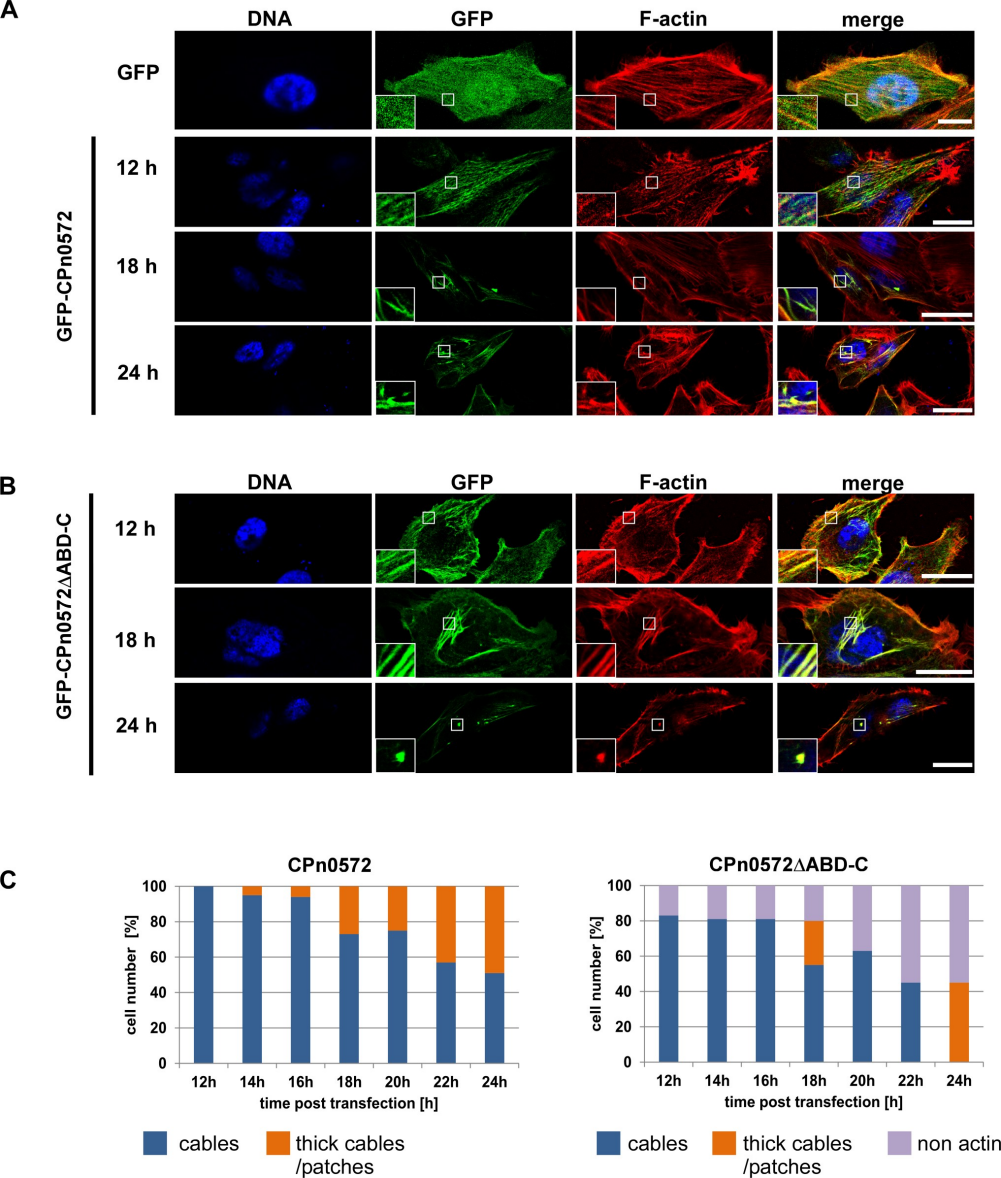
CPn0572ΔABD-C colocalizes with actin in mammalian cells. Confocal images of cells transfected with plasmids encoding GFP or GFP-CPn0572 **(A)** or GFP-CPn0572ΔABD-C **(B)**. HEp-2 cells were transfected with indicated plasmids for 12, 18, or 24 h and F-actin was visualized with phalloidin (red) and DNA with DAPI (blue). Scale bar: 10 μm. Boxed regions show 3-fold enlargement. **(C)** Quantification of GFP-CPn0572 and GFP-CPn0572ΔABD-C-derived phenotypes after transfection, monitored every 2 h (range: 12 to 24 h). n ≥ 100 cells per time point and per transfected plasmid. All quantifications were reproducible and analyzed from triplicates.

We have previously described a drastic reorganization of the actin cytoskeleton in mammalian cells expressing CPn0572 [23]. This strong change in the actin cytoskeleton was observed 24 h post transfection, a time at which standard plasmid expression is high [35]. To visualize early events in the actin cytoskeleton modifications due to CPn0572 expression and thus to identify more subtle actin-related phenotypes, a time-course experiment was carried out in HEp-2 cells transfected with GFP-CPn0572. The GFP signal from control cells was distributed evenly throughout the cell and did not colocalize with phalloidin-stained actin (Fig 3A, top panels). In contrast, GFP-CPn0572 was localized to < 200 nm-thick cable-like actin structures (referred to as “cables” in this study) in all cells observed as early as 12 h post transfection (Fig 3A, panel 2). At this early time point, no abnormal actin structure could be identified. However, 14 h post transfection, and progressively thereafter, 5% of cells expressing GFP-CPn0572 displayed GFP signal in actin positive patches and > 200 nm-thick cable structures (referred to as “thick cables” in this study) (Fig 3A, panel 3; quantified in Fig 3C), structures not found in control cells. At 24 h post transfection, nearly half of the cells displayed this phenotype (Fig 3A, panel 4; quantified in Fig 3C). Thus, CPn0572 binding to actin stimulates the generation of thick actin cables and eventually patches, potentially through direct binding and bundling of actin cables [14] in a time-dependent manner.

In order to test if the formation of these structures was dependent on the ABD-C domain only, we repeated the time-course experiment with the GFP-CPn0572ΔABD-C construct. Compared to full-length CPn0572, which colocalized with actin cables in 100% of cells observed 12 h post transfection, 83% of GFP-CPn0572ΔABD-C transfected cells displayed actin cable colocalization at this time point (Fig 3B, top panels; quantified in Fig 3C). At later time points, GFP-CPn0572ΔABD-C localized exclusively to a variety of abnormal actin structures including patches and thick cables (Fig 3B; panels 2 and 3; quantified in Fig 3C). These results show an ABD-C-independent abnormal actin phenotype triggered by CPn0572ΔABD-C expression, consistent with the phenotype observed in CPn0572ΔABD-C expressing yeast cells. Thus, we conclude that a further actin modulating domain exist in CPn0572 apart from the ABD-C domain.

**Fig 4:**
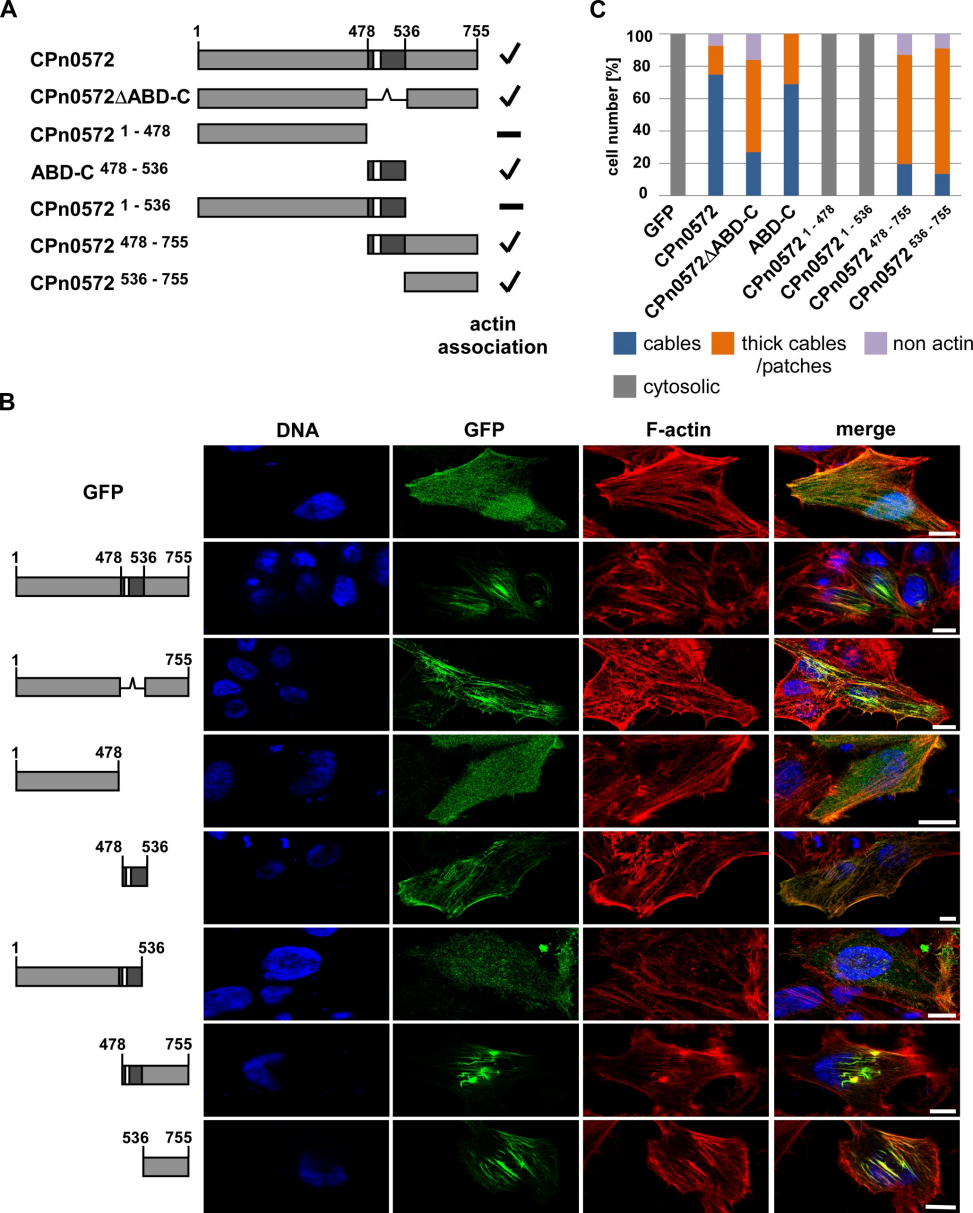
The N-terminus of CPn0572 inhibits actin association of the ABD-C domain while the C-terminus associates with actin in the absence of ABD-C domain. **(A)** Schematic representation of CPn0572 and deletion variants thereof. ABD-C, black box; ABD, white box. Numbers indicate amino acid positions. Positive actin association is indicated by a checkmark and non-association with a minus on the right margin of the drawings. **(B)** Confocal images of HEp-2 cells transfected for 18 h with the indicated CPn0572 variants fused to GFP at the N-terminus. F-actin was visualized with phalloidin (red) and DNA with DAPI (blue). Bar: 10 μm. **(C)** Quantification of intracellular localization of GFP-CPn0572 and variants shown in **(B)**. n ≥ 90 cells per transfected plasmid. All quantifications were reproducible and analyzed from triplicates.

To identify the region of CPn0572 harboring such domain, we generated a battery of plasmids encoding recombinant GFP-fusions of CPn0572 truncations missing either the N-terminus (encoded by amino acids 1–478), the ABD-C domain (encoded by amino acids 478–535), the C-terminus (encoded by amino acids 536–755), or a combination thereof (Fig 4A and S3 Fig), and observed the localization of GFP tagged protein in transfected HEp-2 cells at 18 h post transfection (Fig 4B; quantified in Fig 4C). GFP-CPn0572 and GFP-CPn0572ΔABD-C colocalized with actin structures (Fig 4B, panels 2 and 3), but the localization of the N-terminal GFP-CPn0572^1-478^ variant was cytoplasmic (Fig 4B, panel 4) supporting the notion that the N-terminus did not contain actin binding properties. Importantly, the ABD-C domain of CPn0572 (amino acids 478–536) was sufficient for actin colocalization (Fig 4B, panel 5), demonstrating that this domain is able to interact with actin without the aid of any additional CPn0572-derived domains, reminiscent of the ABD-C actin localization in budding yeast [23]. Surprisingly, when the ABD-C domain of CPn0572 is expressed fused to the N-terminus, the resulting protein GFP-CPn0572^1-536^ did not colocalize with actin, but rather displayed a cytoplasmic distribution (Fig 4B, panel 6). This finding proposes that the N-terminal region (amino acid 1–478) inhibited the ability of the ABD-C domain to bind to actin structures. Interestingly, the C-terminal CPn0572 variants with or without the ABD-C, CPn0572^478-755^ and CPn0572^536-755^ respectively, both colocalized with actin structures (Fig 4B, panels 7 and 8). Thus, an actin binding domain exists downstream of the ABD-C domain. Collectively, our data show that CPn0572^536-755^ harbors an additional actin-binding domain able to modulate the actin cytoskeleton in both yeast and mammalian cells.

### CPn0572^536-755^ binds directly to F-actin

**Fig 5:**
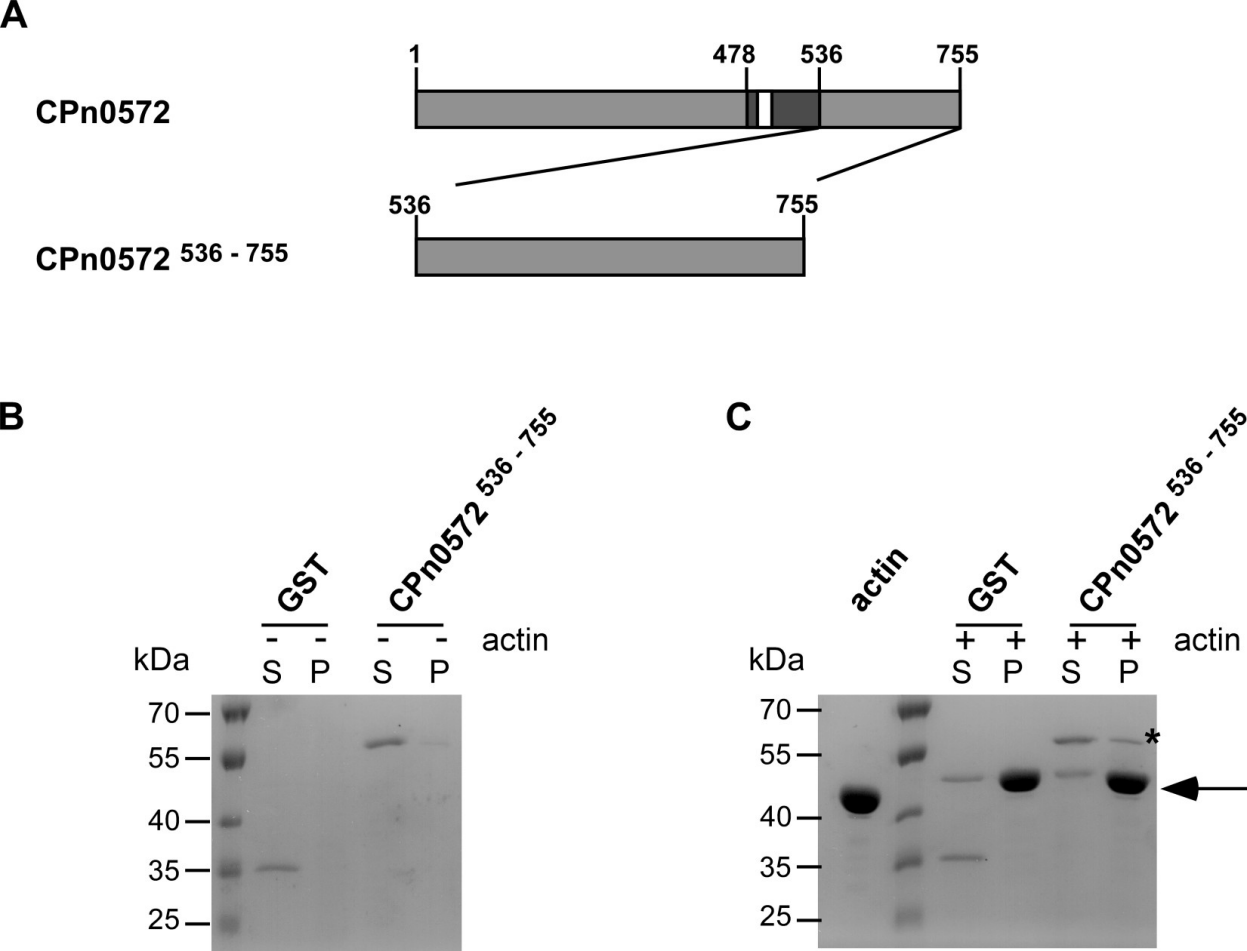
The C-terminus of CPn0572 binds F-actin *in vitro*. **(A)** Schematic representation of CPn0572 and CPn0572^536-755^ variants. ABD-C, black box; ABD, white box; Numbers indicate amino acid positions. **(B)** and **(C)** F-actin binding of CPn0572^536-755^ was analyzed using an actin co-sedimentation assay. Experiments were performed in the absence (-) or in the presence (+) of 40 μg of pre-assembled F-actin (B and C, respectively). Supernatant (S) and pellet (P) fractions were analyzed by SDS-PAGE and visualized with Coomassie blue. Actin is marked with an arrow and GST-CPn0572^536-755^ in the pellet fraction is marked with a (*). n = 2.

In order to further examine the actin binding capacity of the newly identified actin modulatory domain within CPn0572^536-755^, we used bacterially expressed recombinant proteins and tested them in an actin co-sedimentation assay. In short, pre-assembled F-actin was incubated with recombinant GST control or GST-CPn0572^536-755^ (Fig 5A) followed by ultracentrifugation. The ultracentrifugation step generated an F-actin pellet that was analyzed for proteins co-sedimenting along with F-actin. In the absence of actin filaments, both proteins remained exclusively in the soluble fraction (Fig 5B). However, when F-actin was present, a proportion of GST-CPn0572^536–755^ was found to co-sediment with F-actin (Fig 5C). This *in vitro* actin binding analysis shows that a second actin-modulating domain exists within the C-terminus of CPn0572 (amino acids 536–755) that can bind F-actin directly.

### CPn0572^536–755^ harbors actin and vinculin-binding domains

**Fig 6:**
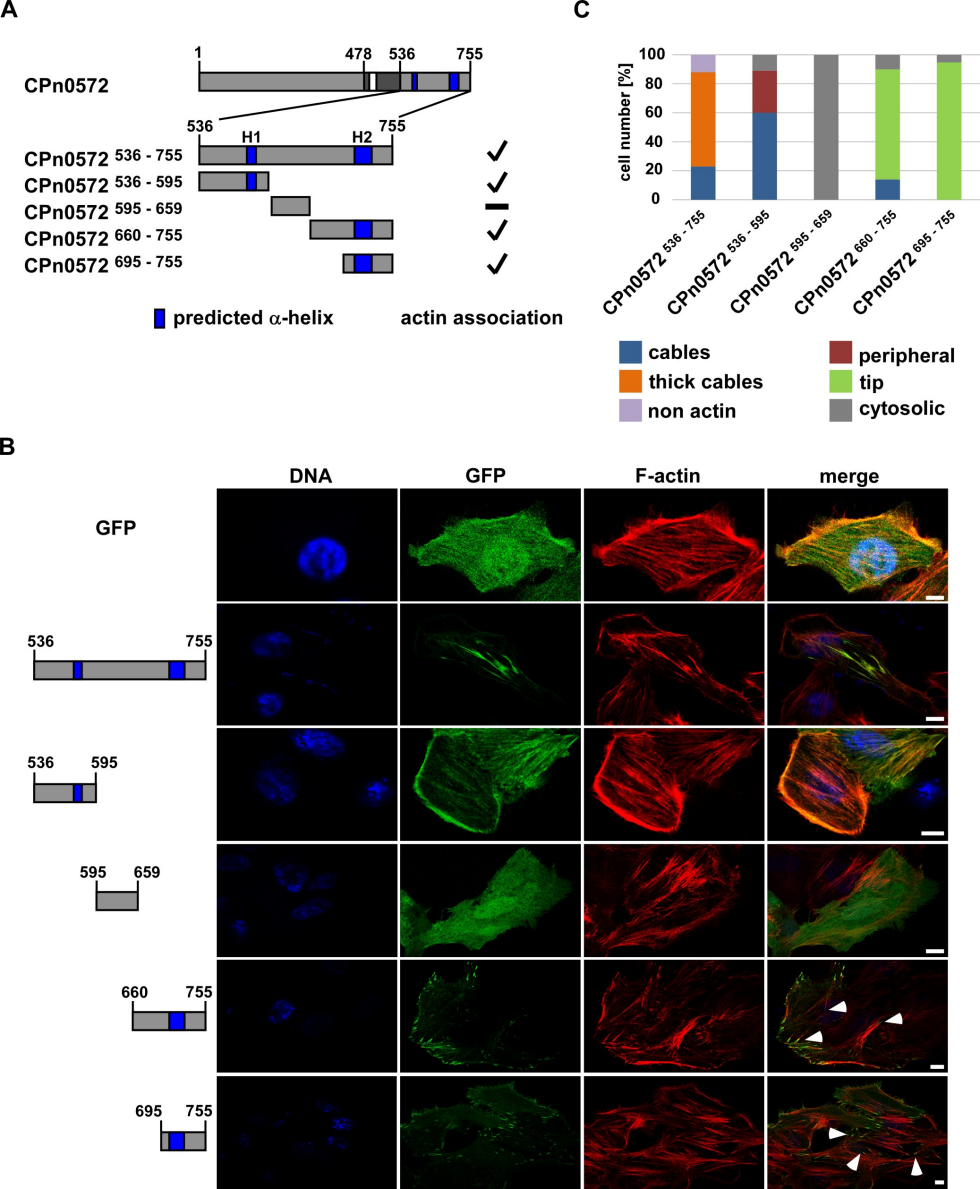
CPn0572^536-595^ associates with actin. **(A)** Schematic representation of CPn0572 and C-terminal variants. ABD-C, black box; ABD, white box; predicted α-helices, dark blue boxes (labeled H1 and H2). Numbers indicate amino acid positions. Positive actin association is indicated by a checkmark and non-actin association with a minus on the right margin of the drawings. **(B)** Confocal fluorescence microscopy analysis of transfected cells expressing GFP or GFP-CPn0572 variants. HEp-2 cells were transfected for 18 h before fixation. F-actin was visualized with phalloidin (red) and DNA with DAPI (blue). Tips of actin cables are marked by white arrow heads. Bar: 10 μm. **(C)** Quantification of localization phenotypes of GFP-CPn0572 and variants shown in **(B)**: CPn0572^536-755^ (n = 572), CPn0572^536-595^ (n = 313), CPn0572^595-659^ (n = 380), CPn0572^660-755^ (n = 347) and CPn0572^695-755^ (n = 563). All quantifications were reproducible and analyzed from triplicates.

To better define the actin modulating domain in CPn0572^536–755^ we performed a secondary-structure prediction analysis and identified candidate alpha-helical structures that could bind actin. A large number of actin binding proteins utilize an alpha-helix to interact within the hydrophobic cleft on the surface of actin, which represents a binding-hot spot for actin modulating proteins [36]. In fact, L2-TarP contains three alpha-helices that allow it to interact with either G-actin or F-actin [37]. Secondary structure elements as calculated by SOPMA [38] revealed two regions predicted to form alpha helices, amino acids 572–581 (Helix 1, referred to as H1 in this study) and amino acids 711–736 (Helix 2, referred to as H2) (S2A Fig). We then tested if protein variants containing these alpha helices would colocalize with actin in HEp-2 cells. Plasmids encoding N-terminal GFP-fusions of CPn0572^536–755^, CPn0572^536–595^, CPn0572^595–659^, CPn0572^660–755^ or CPn0572^695–755^ (Fig 6A and S3 Fig) were expressed in HEp-2-cells, which were fixed and subsequently stained with rhodamine-phalloidin. As shown previously the majority of GFP-CPn0572^536–755^ expressing cells displayed localization of the fusion protein to thick actin cables (Fig 6B, panel 2; quantified in Fig 6C). Interestingly, in 89% of the cells expressing CPn0572^536–595^, which contains H1, the fusion protein localized to actin structures such as actin cables and actin-positive structures at the cell periphery (Fig 6B, panel 3; quantified in Fig 6C), demonstrating that this domain possesses actin-binding properties. In contrast, CPn0572^595–659^, which does not contain either H1 or H2, localized exclusively to the cytoplasm (Fig 6B, panel 4; quantified in Fig 6C). Surprisingly, CPn0572^660–755^ and CPn0572^695–755^, both of which contain the H2 motif, did not localize to actin cables. Instead these fusion proteins were present at tip-like structures at the cell periphery, often at the end of long actin cables (Fig 6B, panels 5 and 6; quantified in Fig 6C). This type of localization is reminiscent of vinculin staining at focal adhesions [39].

Some TarP orthologs have been shown to contain VBSs, which mediate the recruitment of vinculin through direct protein-protein interactions [13, 20, 22]. However, no VBS had been identified for CPn0572 thus far. Our results suggested that CPn0572 might contain a vinculin-binding site present between amino acids 695–755. Indeed, visual analysis of the primary sequence in this region of CPn0572 revealed a predicted VBS at amino acids 717–735 similar to the consensus LxxAAxxVAxxVxxLIxxA [40] (S2B Fig).

**Fig 7:**
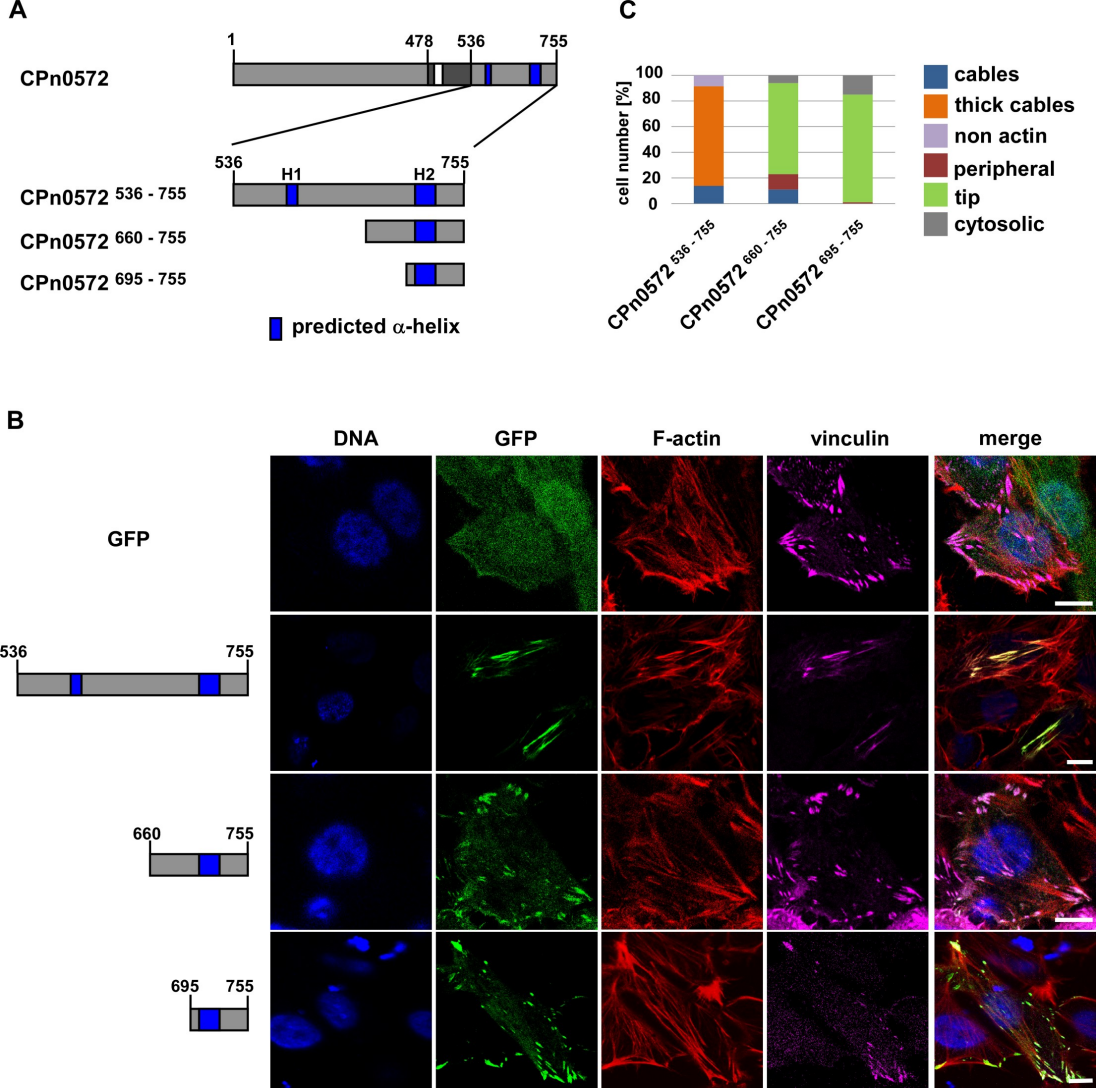
The C-terminus of CPn0572 contains a vinculin-binding domain. **(A)** Schematic representation of CPn0572 with its C-terminal variants. ABD-C, black box; ABD, white box; predicted α-helices, dark blue boxes (labeled H1 and H2). Numbers indicate amino acid positions. **(B)** Confocal fluorescence microscopy analysis of transfected cells expressing GFP or GFP-CPn0572 and vinculin fused to SNAP. HEp-2 cells were transfected for 18 h before fixation. F-actin was visualized with phalloidin (red), vinculin with SNAP-cell SiR647 (magenta) and DNA with DAPI (blue). Bars: 10 μm. **(C)** Quantification of localization phenotypes of GFP-CPn0572 variants shown in **(B).** n ≥ 90 cells per transfected plasmid. All quantifications were reproducible and analyzed from triplicates.

To assess the ability of CPn0572 to colocalize with vinculin, HEp-2 cells were co-transfected with plasmids encoding CPn0572^536-755^, CPn0572^660-755^ or CPn0572^695-755^ fused to GFP at the N-terminus (Fig 7A and S3 Fig) and a plasmid encoding SNAP-tagged vinculin. Cells were labeled with the fluorescent SNAP-Cell SiR substrate and co-stained with phalloidin (Fig 7B; quantified in Fig 7C). In contrast to GFP-expressing control cells, where vinculin was concentrated in puncta at the tip of actin cables near the cell periphery, cells expressing GFP-CPn0572^536-755^ mislocalized vinculin throughout the length of GFP-positive actin cables (Fig 7B, panels 1 and 2). Moreover, vinculin was no longer detected at the cell periphery, suggesting that CPn0572 was able to recruit it to abnormal actin structures. Interestingly, GFP-tagged CPn0572 proteins containing H2, namely GFP-CPn0572^660-755^ and GFP-CPn0572^695-755^, localized to typical vinculin structures, displayed as puncta at the cell periphery, and showed no disruption of the actin cytoskeleton (Fig 7B, panels 3 and 4; quantified in Fig 7C).

**Fig 8:**
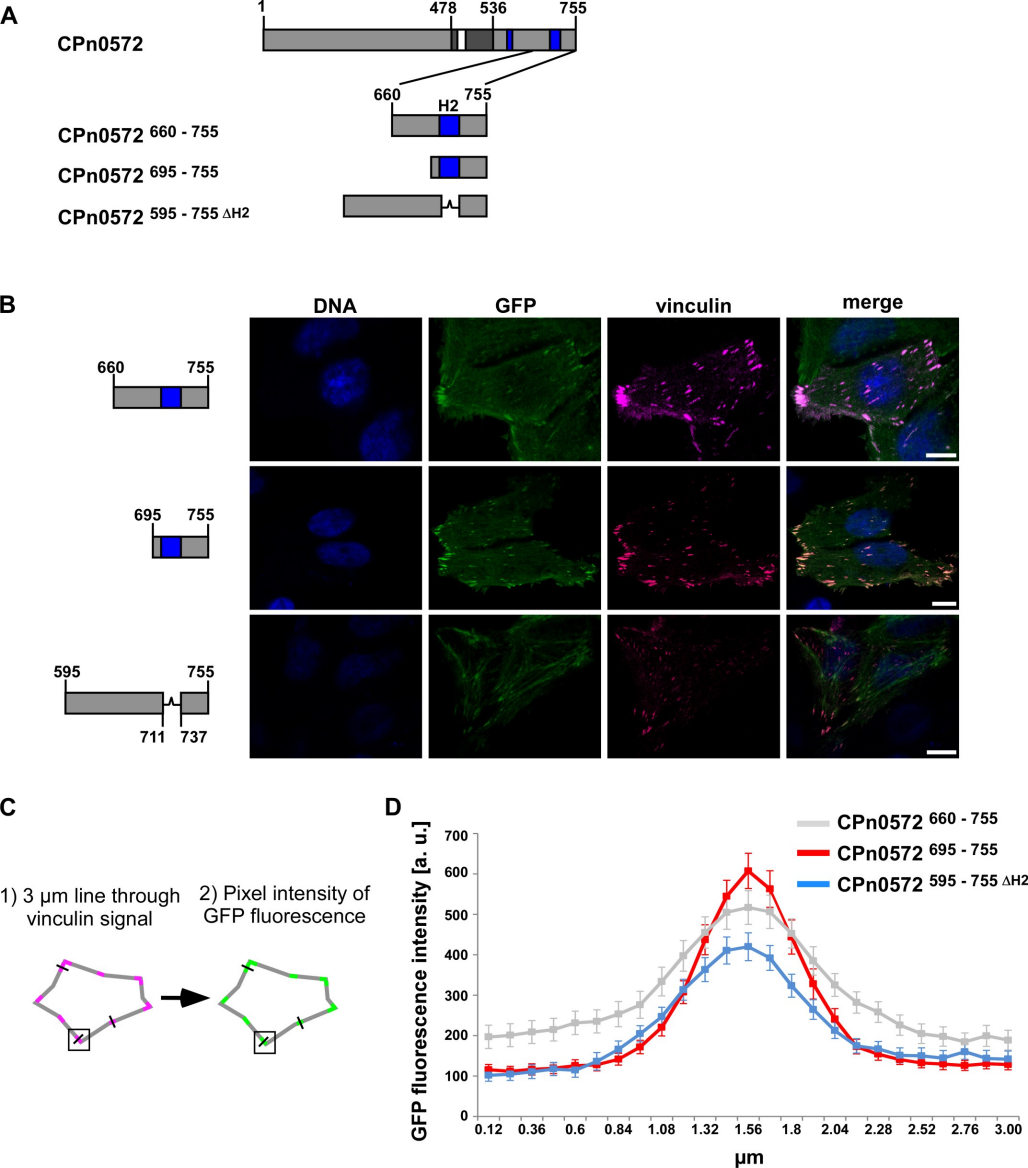
Helix 2 of CPn0572 plays a role in vinculin association. **(A)** Schematic representation of CPn0572 and variants thereof. **(B)** Confocal fluorescence microscopy analysis of transfected cells expressing CPn0572 variants fused to GFP and vinculin fused to SNAP. HEp-2 cells were transfected for 18 h before fixation. Vinculin was visualized with SNAP-cell SiR647 (magenta) and DNA with DAPI (blue). Bars: 10 μm. **(C)** Schematic representation of the methodology used for the quantification of GFP fluorescence intensity colocalized with vinculin. **(D)** GFP fluorescence intensity of GFP-CPn0572 variants that colocalize with vinculin puncta. arbitrary units (a.u.). n = 90 independent measurements for each construct analyzed from triplicates.

To determine if the predicted VBS of CPn0572 is required for vinculin association, we generated a plasmid encoding GFP-CPn0572^595–755ΔH2^, in which the VBS within H2 was deleted (Fig 8A) and transfected HEp-2 cells (S3A and S3C Fig). Compared to GFP-CPn0572^660-755^ and GFP-CPn0572^695-755^, the colocalization of GFP-CPn0572^595-755ΔH2^ and vinculin was reduced (Fig 8B, bottom panels; quantified in Fig 8D), demonstrating that H2 is important for vinculin-binding. However, a weak GFP-CPn0572^595-755ΔH2^ signal could still be detected colocalizing with vinculin staining. Further visual analysis of the amino acid sequence in CPn0572^595–755^ revealed the presence of a second sequence upstream of the H2, with a lower homology to the consensus VBS and the CPn0572 VBS, at amino acids 682–700 (S2C Fig). The residual vinculin colocalization observed for GFP-CPn0572^595–755ΔH2^ could be a result of the presence of this second VBS-like sequence.

Taken together, our data have led to the identification of a new CPn0572 actin-binding domain at the proximal end of the C-terminal domain (amino acids 536–595) and a vinculin-binding domain at the end of the C-terminus (amino acids 717–736). Thus, we propose that the reorganization of the actin cytoskeleton by the *C*. *pneumoniae* TarP family member is driven by a combination of the ABD-C domain, the F-actin binding domain and the vinculin binding domain.

## Discussion

In this study we have used the fission yeast *S*. *pombe* to confirm that the ABD-C domain of CPn0572 has strong actin cytoskeleton remodeling capacity. Moreover, the yeast analysis revealed that CPn0572 must contain an ABD-C independent actin modulatory domain. We recapitulated these findings in human epithelial HEp-2 cells and mapped an additional actin modulatory domain to amino acids 536–595 of CPn0572. Indeed, the C-terminus of CPn0572 bound F-actin directly *in vitro*, thus revealing it to harbor an FAB domain. Moreover, we demonstrated that the N-terminus of CPn0572 is able to inhibit the ability of the ABD-C domain to aggregate actin in HEp-2 cells. Finally, our results also identify a VBS in CPn0572, which enables it to colocalize with vinculin and recruit it to actin cables decorated with CPn0572.

An attractive advantage of utilizing fission yeast in the study of chlamydial effectors is the simplicity of the system. For instance, fission and budding yeast do not have many of the signaling pathways involved in *Chlamydia* entry, nor do they encode proteins involved in focal adhesion like vinculin. As a result, we can observe the impact of TarP on actin reorganization without the complexity of other pathways regulating its function. As a result, fission yeast represents an excellent tool for exploring domain structure-function relationships.

In *Chlamydia* species the TarP family has been established as a master regulator of actin dynamics during bacterial invasion. Members of this family have the ability to bind actin directly through a number of actin binding sequences and the capacity to interact with a number of cellular host factors that mediate cell signaling and participate in actin reorganization [13–16, 20, 21, 23, 37] (domains identified thus far in *C*. *trachomatis* TarP and *C*. *pneumoniae* CPn0572 are summarized in S4 Fig). In this study, we show that expression of full length CPn0572 in fission yeast or in mammalian cells results in a strong reorganization of the actin cytoskeleton; a phenotype that intensifies the longer CPn0572 is expressed. We and others have previously shown that *C*. *pneumoniae* CPn0572 possesses a single ABD motif able to bind actin [14, 23]. For this assay, Jewett et al. utilized a 100-amino acid-long region of CPn0572, which includes a 12 amino acid-long motif with high sequence identity to TarP family ABDs, embedded in an 19 amino acid-long alpha helix [14], which is proposed to be the G-actin-binding motif of TarP [37]. Interestingly however, we now show that expression of an ABD deletion version of CPn0572 (CPn0527ΔABD-C) exhibits a clear impact on the actin cytoskeleton in both yeast and human model systems, which suggested the presence of additional actin modulatory regions fully capable of altering the actin cytoskeleton in both systems. Subsequently, we found that the C-terminus of CPn0572 (amino acids 536–755) can by itself remodel the actin cytoskeleton and harbors an FAB domain.

Members of the TarP family can have up to three functional ABDs [14]. In addition to the ABDs, two FABs were identified experimentally in *C*. *trachomatis* L2 TarP. Sequence comparisons identified FABs in other *C*. *trachomatis* serovars and potential FABs in TarP orthologs from different chlamydial species; however, such domains were not identified in the *C*. *pneumoniae* TarP ortholog [21]. Our study demonstrated that CPn0572 contains at least one ABD and one FAB, consistent with the notion that TarP family members depend on ABDs and FABs to mediate the cytoskeletal changes to facilitate chlamydial invasion. The interplay between these domains during chlamydial entry is as yet unknown, but likely requires spatiotemporal regulation of the various actin modulatory domains present in the same molecule. However, it is possible that these domains have roles in separate steps of the chlamydial infection cycle.

Interestingly, the N-terminus of CPn0572, like the TarP orthologs of *C*. *caviae*, *C*. *abortus*, *C*. *felis* and *C*. *muridarum*, does not contain the tyrosine-rich repeats identified in *C*. *trachomatis* [13] and, as of yet, no function has been attributed to N-terminus of these orthologs. We now present preliminary evidence that the N-terminal domain of CPn0572 (amino acids 1–478) inhibits the actin-recruiting capacity of the ABD-C domain. The ABD-C of CPn0572 clearly localizes to actin structures in mammalian cells and budding yeast [23]. Addition of the CPn0572 N-terminus to the ABD-C abrogates actin localization in mammalian cells but not in budding yeast [23]. This suggests that the regulation of the ABD-C by the N-terminus may require mammalian host factors. The inhibitory effect of the N-terminus appeared to be specific for the ABD-C, as the C-terminal construct, amino acid 536–755, localized to actin in the presence of the N-terminus. Furthermore, the N-terminus in the context of full-length CPn0572 does not inhibit the protein from localizing to actin structures, uncovering an essential role for the C-terminus (amino acids 536–755) this process. It is likely that the ability of the N-terminus to regulate the ABD-C is itself regulated during infection. Interestingly, a *C*. *trachomatis* TarP construct containing the N-terminus but missing the C-terminal ABD and FAB domains still localizes to puncta, albeit without actin colocalization [16] whereas a similar CPn0572 construct localizes throughout the cytoplasm. This observation suggests that there are important functional differences in how the N-terminus of these proteins contributes to the localization of *C*. *trachomatis* TarP and CPn0572, possilby due to the presence of the tyrosine-rich domain in *C*. *trachomatis* TarP that is missing in CPn0572.

Importantly, we also identify a vinculin-binding domain (VBD) in the C-terminus of CPn0572 containing a 19-residue-long motif characteristic of a VBS. Multiple VBSs have been identified in the *Shigella* protein IpaA and the *Rickettsia* protein Sca4 [41], and both effector proteins are thought to activate vinculin to mediate localized actin remodeling. In *Chlamydia*, RNA interference screens identified the necessity of vinculin during *C*. *trachomatis* L2 infection in culture cells [42, 43]. Additionally, vinculin was found to be recruited to sites of *C*. *caviae* entry while infection efficiency was reduced in vinculin KO cells, revealing that vinculin plays a role during invasion of *C*. *caviae* [13]. The precise role of vinculin during *Chlamydia* infection and whether it cooperates with the ABDs and FABs of the TarP family during invasion remains unknown at present. However, the VBD in CPn0572 is likely to be implicated in the recruitment of actin or the remodeling of the actin cytoskeleton. In mammalian cells, CPn0572 C-terminus containing both the FAB and the VBS localizes mostly to thick actin cables whereas a CPn0572 C-terminus construct containing only the FAB localizes to thin actin cables and the cell periphery. Vinculin recruitment to CPn0572 could enhance formation of thick actin cables through vinculin’s actin and Arp2/3 binding domains [44]. Additionally, the lack of actin localization of CPn0527ΔABD-C in fission yeast pointed to additional mammalian-specific factors that stabilize the FAB domain interaction with actin. Vinculin could be one such factor. Fission yeast does not possess a vinculin homologue. Thus, the CPn0572 VBS could not aid in the formation of actin structures in this organism. These observations are consistent with recent findings in which the VBD of *C*. *caviae* TarP was sufficient for the recruitment of actin, but this ability was abrogated in vinculin KO cells [13]. Interestingly, as opposed to the VBD of *C*. *caviae* TarP, the VBD of CPn0572 was not sufficient to mediate recruitment of actin.

The data presented here demonstrate that the TarP family member CPn0572 has potential cytoskeletal remodeling functions at its C-terminal through vinculin and actin binding and recruitment. The N-terminal domain has a negative impact on the ABD actin binding. Further work will be necessary to elucidate the timing and specific roles of these domains during *C*. *pneumoniae* infection.

## Material and methods

### Cloning procedures, vector constructs and antibodies

Full length CPn0572 and variants were amplified by PCR from pre-existing vectors with custom synthesized oligonucleotide primers (Sigma Aldrich). Plasmids were generated by amplification of DNA fragments that were subsequently cloned into pJR1-3XU with or without mCherry (C-terminal mCherry) for yeast expression, pAE67 (CMVp N-terminal GFP) for mammalian expression vector or pKM36 (GST-Tag) for expression in *E*. *coli*. All constructs were generated by homologues recombination in *S*. *cerevisiae*. The SNAP-tag fusion plasmid vinculin-23 was a gift from Michael Davidson (Addgene plasmid #58198). Antibodies against mCherry (Abcam), GFP (chromotek) and γ-tubulin (Sigma) as well as secondary antibodies anti-rabbit (Promega) and anti-mouse (Sigma) were used for western blots.

### Yeast strains and media

Wild-type, (UFY605: *his3-D1*, *ade6-M210*, *leu1-32*, *ura4-D18*, *h*^*-*^) a gift from K. Gould, Vanderbilt University, USA, or lifeact-GFP, (UFY2999: *Pact1-LAGFP*::*leu1*^*+*^, *ura4-D18*, *leu1-32 h*^*-*^) a gift of M. Balasubramanian, Warwick Medical School, UK, were used to obtained transformants by introducing plasmids in cells grown in YE5S and later maintained in minimal media with supplements as described [45]. To repress or derepress expression, cells bearing *nmt1*^*+*^ promoter plasmids were grown in minimal medium plus 5 μg/ml thiamine for at least 48 h, washed twice with thiamine-less medium and finally resuspended in either media with thiamine or thiamine-less medium for 22 h at 30°C and either utilized for serial-dilution patch tests, live-cell microscopy or preparation of protein extracts. For patch test analysis, transformants were spotted in 10-fold serial dilutions in plates containing 1 μM or 2 μM Latrunculin B (Merck Chemicals, Germany), or DMSO. For western blots, 100 OD_600_ units of cells were used to prepare protein extracts, as described previously [46].

### Live cell microscopy of fission yeast

For imaging of living *S*. *pombe* cells, cells were pre-grown in minimal plasmid-selection medium at 30°C to an OD_600_ of 0.5 and stained with either Hoechst 33342 for 30 minutes or calcofluor white for 5 minutes. Cells were directly processed for microscopy by mounting them on slides. Images of live cells were acquired with a Zeiss Axiovert 200 fluorescence microscope (Carl Zeiss) using a 63x objective with a charge-coupled-device (CCD) camera (IEEE1394-Based Digital Camera Orca-ER 1394; Hamamatsu, Herrsching, Germany) and image editing and analysis was done with ImageJ 1.47v (National Institutes of Health). GFP and mCherry signals were exposed the same amount of time and processed equally for all strains in a given experimental condition.

### Maintenance, transfection and staining of mammalian cells

HEp-2 cell were cultured in DMEM medium supplemented with 10% fetal calf serum (FCS), MEM vitamins (Thermo Fisher Scientific) and non-essential amino acids (Thermo Fisher Scientific) and incubated at 37°C and 6% CO_2_. A 40% confluent HEp-2 cell monolayer was grown on cover slips in 24 well plates (Sarstedt). For transfections, cells in fresh medium without FCS were transfected for 18 h using TurboFect (Thermo Fisher Scientific). Transfected HEp-2 cells were fixed with 3% paraformaldehyde in PBS for 10 min, washed three times with HBSS and permeabilized with 2% saponin (Sigma Aldrich) in PBS for 20 min at 30°C. Staining of actin in human cells with rhodamine-phalloidin was performed as recommended by the manufacturer (Thermo Fischer Scientific). DAPI was used to visualize DNA and the far-red fluorescent substrate SNAP-Cell 647-SiR (New England Biolabs) to visualize SNAP-tagged Vinculin.

### Microscopy of mammalian cells and image processing

Images were acquired using an inverse Nikon TiE Live Cell Confocal C2plus with 100 x TIRF objective and a C2 SH C2 Scanner. Analysis of images and measurements were generated with Nikon Element software. The thickness threshold under which long fibrous phalloidin-positive actin structures were identified as “cables” and above which long fibrous actin structures were identified as “thick cables” was set at 200 nm. Images for analysis of actin cable thickness was done using ImageJ 1.47v (National Institutes of Health). Quantification of CPn0572-derived phenotypes after transfection in HEp-2 cells was done by classifying individual cells in described phenotypic groups utilizing the GFP signal.

### Quantification of GFP fluorescence intensity that colocalizes with vinculin fluorescence signal

For quantification of fluorescence intensity of GFP-CPn0572 variants at sites of vinculin localization, we transfected HEp-2 cells with SNAP-tagged vinculin and the relevant GFP constructs, stained cells with far-red fluorescent substrate SNAP-Cell 647-SiR, and captured images by confocal microscopy. GFP and vinculin signals were acquired with identical microscope settings. Quantification was done with ImageJ 1.47v (National Institutes of Health) as follows: For each cell analyzed, three 3 μm lines were drawn through individual vinculin puncta. The middle of the line was kept at the center of each vinculin signal. The line was used to measure the pixel intensity of the GFP fluorescence every 0.12 μm for a total of 25 measuring points per line. For each of the constructs the background signal was determined by drawing 8 lines in areas without cells. The signal intensity for the background was homogenous and accounted for less than 0.2% of the fluorescence intensity measured in the lines drawn through GFP-CPn0572 expressing cells. The average background signal was subtracted from each of the data points. A total of 90 lines representing 30 cells were analyzed for each GFP construct.

### Protein purification

GST-tagged proteins were expressed in *E*. *coli* BL21 and purified under native conditions from bacterial cell lysate by affinity chromatography using columns with Pierce Glutathione agarose (Thermo Fisher Scientific) and performed as recommended by the manufacturer. Proteins were eluted with reduced glutathione (Roth). Protein concentration was calculated by Bradford assay (Bio Rad) and verified using SDS/PAGE coupled with coomassie staining.

### F-actin co-sedimentation assay

F-actin was assembled *in vitro* as recommended by the supplier (Cytoskeleton Inc.). 40 μl of 1 μg/μl assembled F-actin was incubated with 5 μg of bacterially purified recombinant GST-tagged protein or GST control in PBS 20% glycerol w/v and incubated for 30 minutes at room temperature. Samples were then spun at 100.000 x g for 1hr. Supernatants were separated from the pellet fraction and analyzed by SDS-gel electrophoresis followed by coomassie staining.

### Bioinformatic analysis

Secondary structure prediction of CPn0572 variants was carried out with SOPMA (https://npsa-prabi.ibcp.fr/cgi-bin/npsa_automat.pl?page=/NPSA/npsa_sopma.html).

### Accesssion numbers

*Chlamydia trachomatis* L2: NC_010287.1

*Chlamydia pneumoniae* GiD: LN847009.1

## Supporting information

S1 Fig Expression of CPn0572 in *S*. *pombe* results in aberrant cell morphology and cytokinesis defects. Examples of lifeact-GFP-expressing cells harboring an mCherry control plasmid or CPn0572-mCherry plasmid grown for 22 h under plasmid selective conditions without thiamine (high expression of CPn0572). In control cells, actin is present in actin patches at cell tips (arrow heads), the contractile ring in the center of dividing cells (*) and actin cables (arrows). Examples of normal (cells 1 and 2) and abnormal (cells 3–5) cell morphology used for quantification in Fig 1C. Cells were stained with Hoechst to visualize DNA. Bars, 5 μm. **(B)** Live cell images of lifeact-GFP-expressing strains to visualize F-actin (green in merged images) harboring indicated mCherry plasmids (red in merged images) grown for 22 h under plasmid selective conditions without thiamine (high expression of CPn0572). Cells were stained with calcofluor white to observe growth zones (blue in merged images). Abnormal accumulation of cell wall material in puncta (white arrow in calcofluor panels, repeated in lifeact-GFP and merged images), abnormal deposition of cell wall material at the cell middle (arrow head in calcofluor panels, repeated in lifeact-GFP and merged images. Bars, 5 μm. **(C)** Quantification of aberrant cell wall deposition at the cell middle as shown in **(B)**. n = 4 samples each representing 20–70 cells. Error bars denote standard error of the mean. Student’s t-test was used to reveal statistical significance. p < 0.005 (**), p < 0.05 (*), and not significant (ns). **(D)** Expression of mCherrry, CPn0572-mCherry and CPn0572ΔABD-C-mCherry in transformed yeast cells grown for 22 h under plasmid selective conditions leading to either low expression (Low) or high expression (High). Western blot was probed with anti-mCherry or anti- γ-tubulin antibodies. mCherrry containing-proteins are marked with (*). As mCherry-tagged proteins were expressed at low levels in the presence of thiamine, we loaded 6x times more protein to detect a signal.
(TIF)

S2 Fig Secondary structure prediction of the CPn0572 C-terminus reveals potential α-helical structures and a vinculin-binding motif. **(A)** Secondary structure prediction carried out with SOPMA. The predicted α-helices are shown as a sequence of blue *h* letters below the amino acid sequence or as dark blue boxes in the schematic representation of CPn0572 and CPn0572 C-terminus (CPn0572^536-755^). Letter *e* stands for extended strand, *c* stands for random coil and *t* for beta turn. **(B)** and **(C)** Schematic representation of CPn0572^536-755^. Predicted α-helices are shown in dark blue. The amino acid sequence of the second predicted α-helix is shown in dark blue and the vinculin-binding motif is highlighted in green. H2 amino acids with identity or high similarity to the vinculin-binding motif sequence are depicted in bold. **(C)** A second possible vinculin-binding motif is underlined in the amino acids sequence. Amino acids in this sequence with identity or high similarity to the vinculin-binding motif sequence are depicted in bold.
(TIF)

S3 Fig Expression of CPn0572 variants. **(A-B)** Schematic representation of the CPn0572 variants analyzed in **(C)** and **(D). (C-D)** Western blot analysis of GFP-CPn0572 and variants. After 18 h transfection GFP and GFP-tagged proteins were analyzed on SDS-PAGE and visualized with an anti-GFP antibody. γ-tubulin was used as a loading control. n = 3 independent transfections per construct.
(TIF)

S4 Fig CPn0572 has a similar domain distribution to *C*. *trachomatis* TarP. Schematic representation of *C*. *trachomatis* TarP L2 and *C*. *pneumoniae* CPn0572. The N-terminal tyrosine (Y)-rich repeat region of *C*.*trachomatis* TarP is not present in CPn0572. For CPn0572, the newly identified FAB domain is depicted in purple and VBS in green. Matching domains in TarP L2 are displayed.
(TIF)
