# Supplementary figures and images for: CPn0572, the C. pneumoniae ortholog of TarP, reorganizes the actin cytoskeleton via a newly identified F-actin binding domain and recruitment of vinculin
Source: PLoS One. 2019 Jan 10;14(1):e0210403. doi: 10.1371/journal.pone.0210403 (PMC6328165; doi:10.1371/journal.pone.0210403)

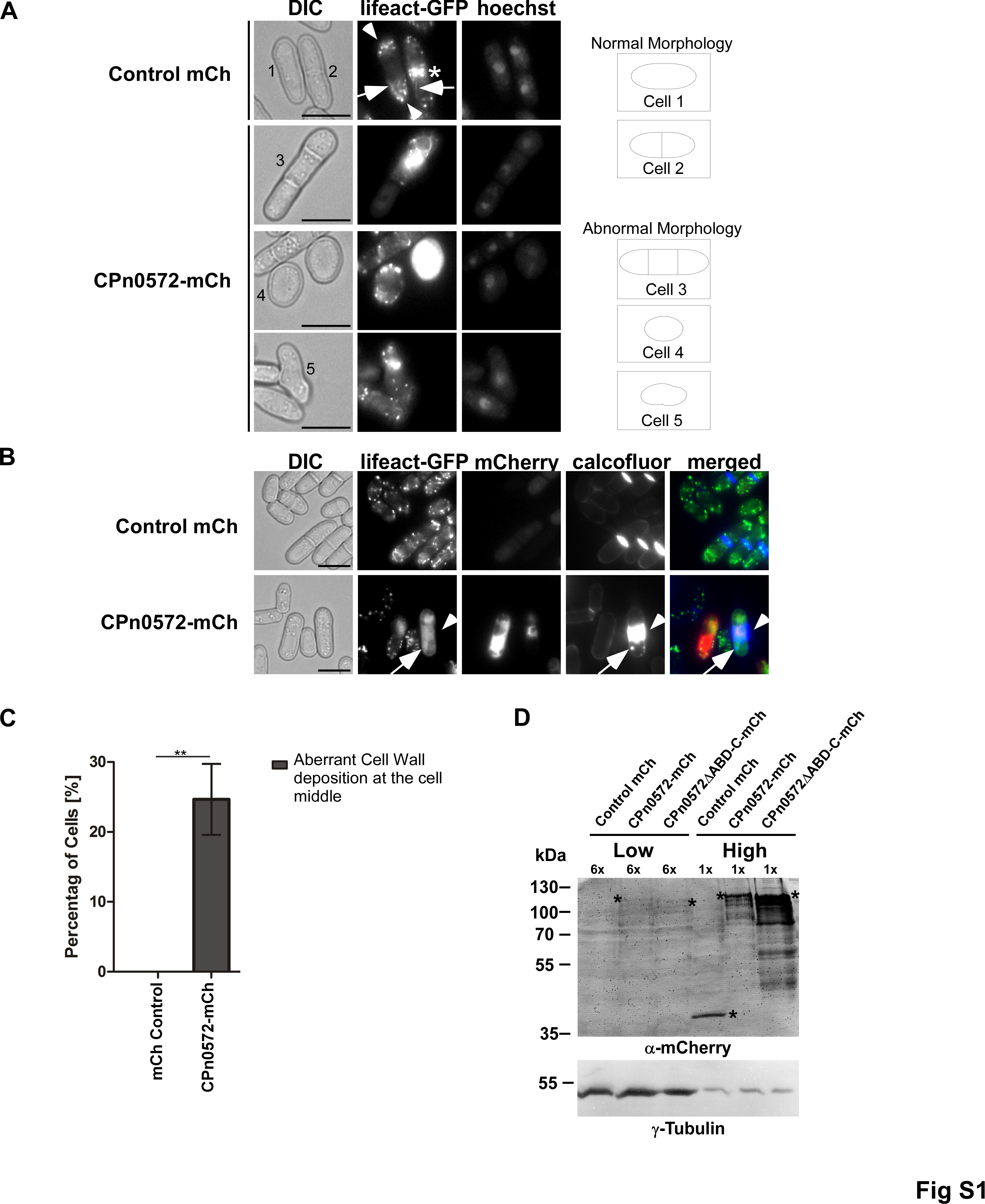

Supplement: S1 Fig — Examples of lifeact-GFP-expressing cells harboring an mCherry control plasmid or CPn0572-mCherry plasmid grown for 22 h under plasmid selective conditions without thiamine (high expression of CPn0572). In control cells, actin is present in actin patches at cell tips (arrow heads), the contractile ring in the center of dividing cells (*) and actin cables (arrows). Examples of normal (cells 1 and 2) and abnormal (cells 3–5) cell morphology used for quantification in Fig 1C. Cells were stained with Hoechst to visualize DNA. Bars, 5 μm. (B) Live cell images of lifeact-GFP-expressing strains to visualize F-actin (green in merged images) harboring indicated mCherry plasmids (red in merged images) grown for 22 h under plasmid selective conditions without thiamine (high expression of CPn0572). Cells were stained with calcofluor white to observe growth zones (blue in merged images). Abnormal accumulation of cell wall material in puncta (white arrow in calcofluor panels, repeated in lifeact-GFP and merged images), abnormal deposition of cell wall material at the cell middle (arrow head in calcofluor panels, repeated in lifeact-GFP and merged images. Bars, 5 μm. (C) Quantification of aberrant cell wall deposition at the cell middle as shown in (B). n = 4 samples each representing 20–70 cells. Error bars denote standard error of the mean. Student’s t-test was used to reveal statistical significance. p < 0.005 (**), p < 0.05 (*), and not significant (ns). (D) Expression of mCherrry, CPn0572-mCherry and CPn0572ΔABD-C-mCherry in transformed yeast cells grown for 22 h under plasmid selective conditions leading to either low expression (Low) or high expression (High). Western blot was probed with anti-mCherry or anti- γ-tubulin antibodies. mCherrry containing-proteins are marked with (*). As mCherry-tagged proteins were expressed at low levels in the presence of thiamine, we loaded 6x times more protein to detect a signal. (TIF) [file pone.0210403.s001.tif]

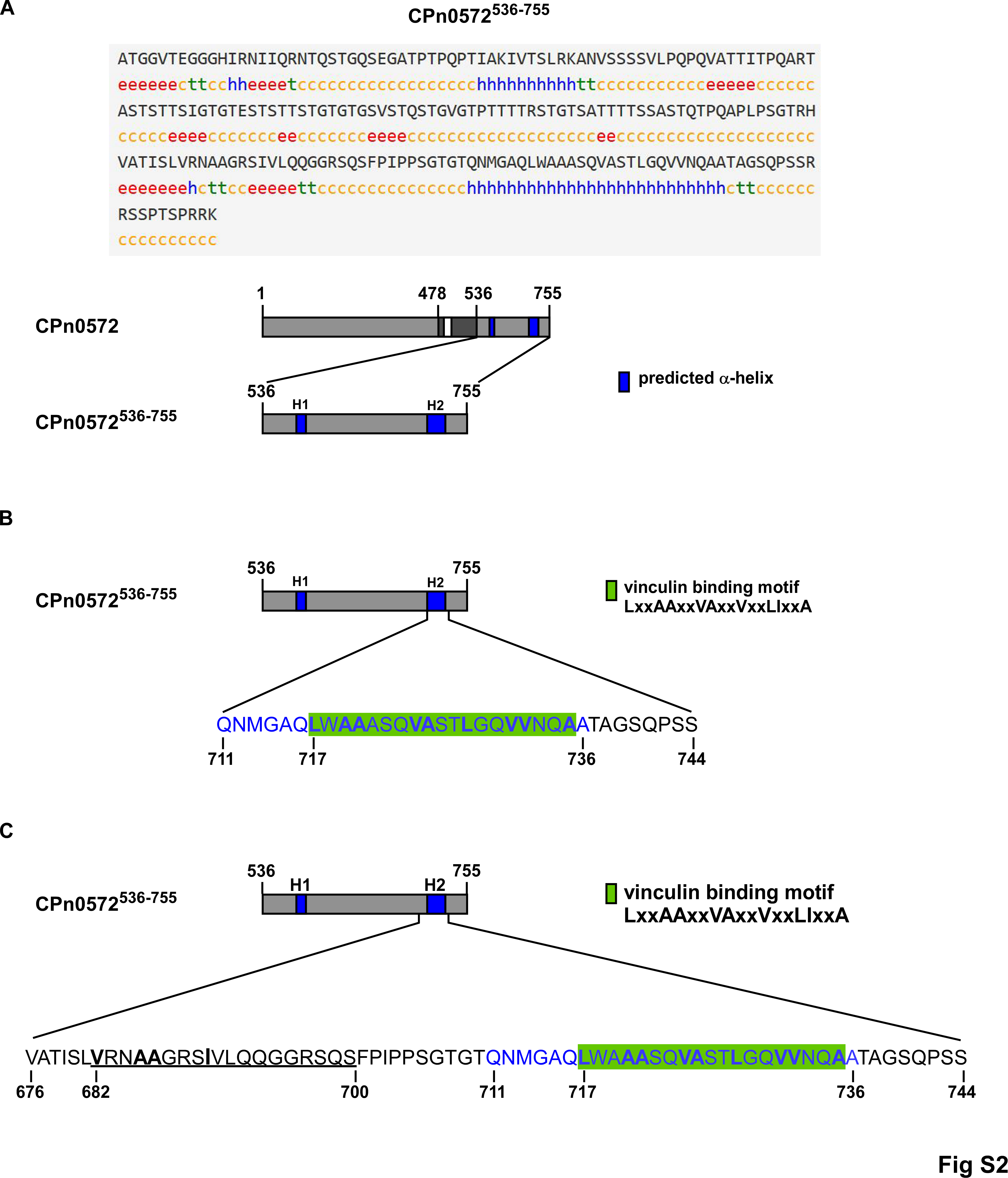

Supplement: S2 Fig — (A) Secondary structure prediction carried out with SOPMA. The predicted α-helices are shown as a sequence of blue h letters below the amino acid sequence or as dark blue boxes in the schematic representation of CPn0572 and CPn0572 C-terminus (CPn0572536-755). Letter e stands for extended strand, c stands for random coil and t for beta turn. (B) and (C) Schematic representation of CPn0572536-755. Predicted α-helices are shown in dark blue. The amino acid sequence of the second predicted α-helix is shown in dark blue and the vinculin-binding motif is highlighted in green. H2 amino acids with identity or high similarity to the vinculin-binding motif sequence are depicted in bold. (C) A second possible vinculin-binding motif is underlined in the amino acids sequence. Amino acids in this sequence with identity or high similarity to the vinculin-binding motif sequence are depicted in bold. (TIF) [file pone.0210403.s002.tif]

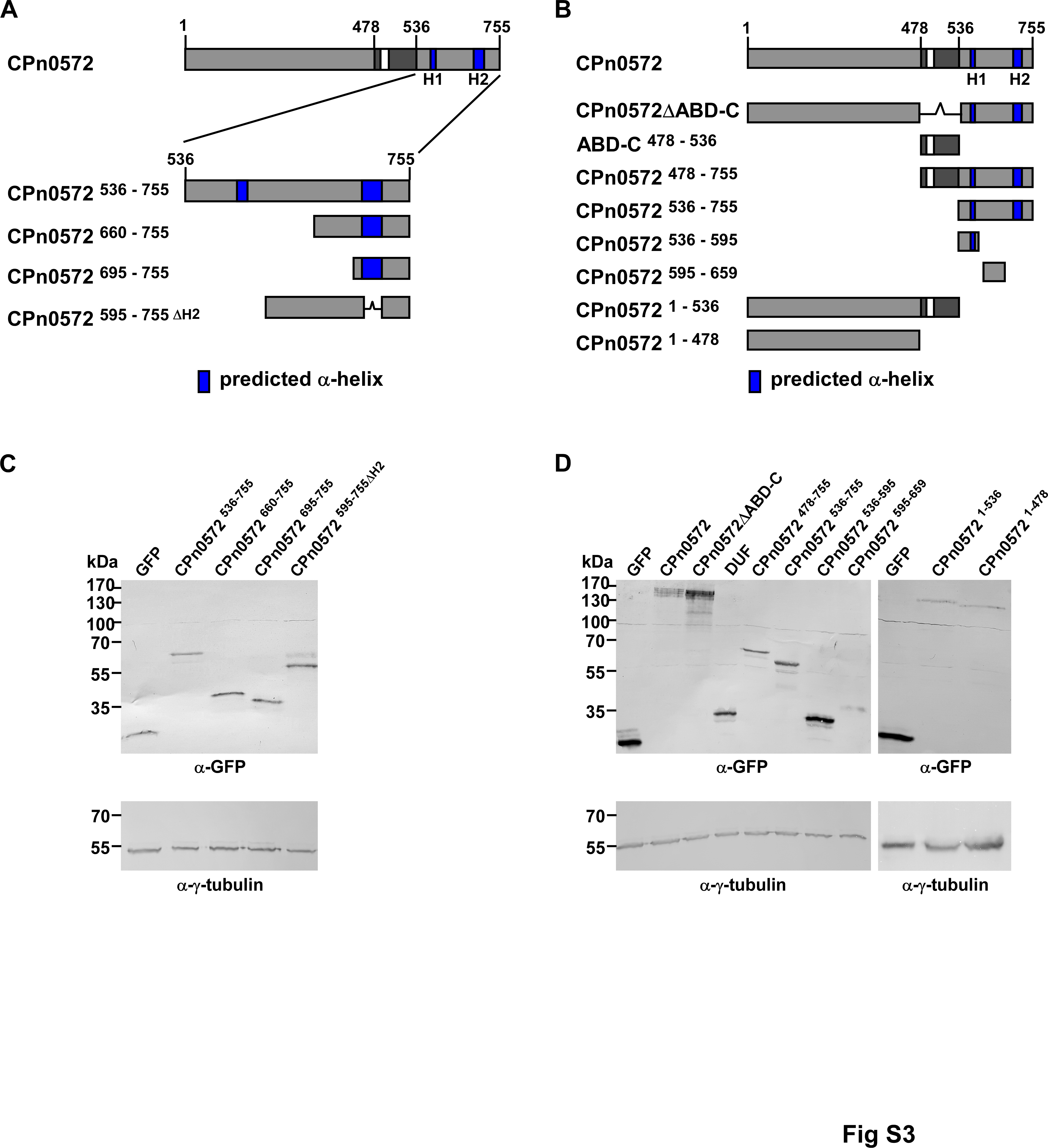

Supplement: S3 Fig — (A-B) Schematic representation of the CPn0572 variants analyzed in (C) and (D). (C-D) Western blot analysis of GFP-CPn0572 and variants. After 18 h transfection GFP and GFP-tagged proteins were analyzed on SDS-PAGE and visualized with an anti-GFP antibody. γ-tubulin was used as a loading control. n = 3 independent transfections per construct. (TIF) [file pone.0210403.s003.tif]

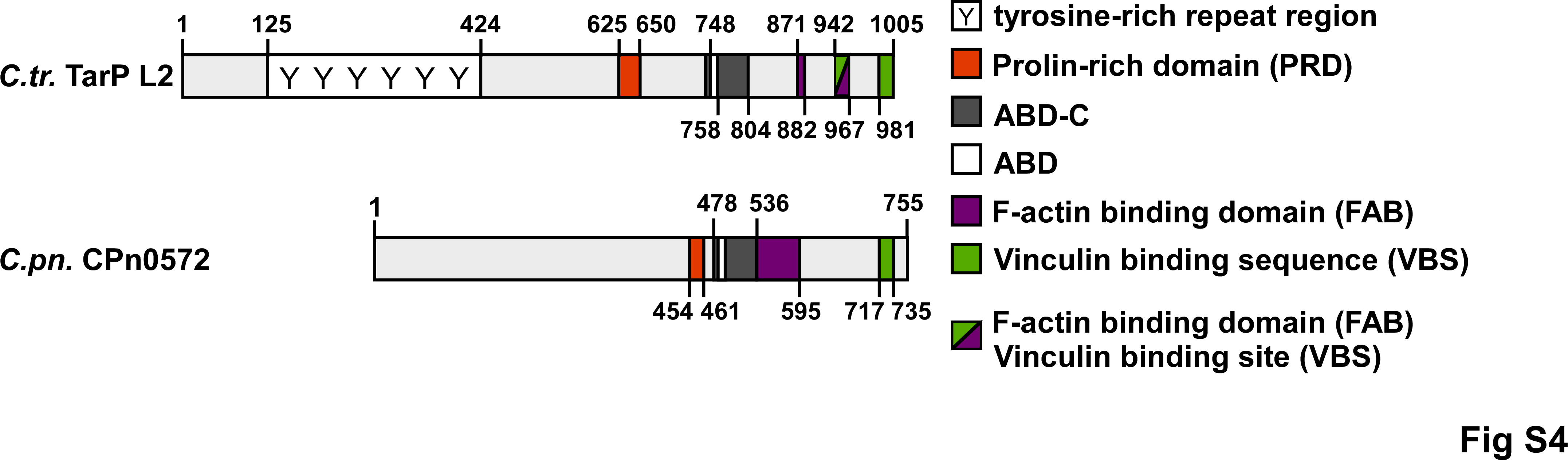

Supplement: S4 Fig — Schematic representation of C. trachomatis TarP L2 and C. pneumoniae CPn0572. The N-terminal tyrosine (Y)-rich repeat region of C.trachomatis TarP is not present in CPn0572. For CPn0572, the newly identified FAB domain is depicted in purple and VBS in green. Matching domains in TarP L2 are displayed. (TIF) [file pone.0210403.s004.tif]
